# Survival by colon cancer stage and screening interval in Lynch syndrome: a prospective Lynch syndrome database report

**DOI:** 10.1186/s13053-019-0127-3

**Published:** 2019-10-14

**Authors:** Mev Dominguez-Valentin, Toni T. Seppälä, Julian R. Sampson, Finlay Macrae, Ingrid Winship, D. Gareth Evans, Rodney J. Scott, John Burn, Gabriela Möslein, Inge Bernstein, Kirsi Pylvänäinen, Laura Renkonen-Sinisalo, Anna Lepistö, Annika Lindblom, John-Paul Plazzer, Douglas Tjandra, Huw Thomas, Kate Green, Fiona Lalloo, Emma J. Crosbie, James Hill, Gabriel Capella, Marta Pineda, Matilde Navarro, Joan Brunet Vidal, Karina Rønlund, Randi Thyregaard Nielsen, Mette Yilmaz, Louise Laurberg Elvang, Lior Katz, Maartje Nielsen, Sanne W. ten Broeke, Sigve Nakken, Eivind Hovig, Lone Sunde, Matthias Kloor, Magnus v Knebel Doeberitz, Aysel Ahadova, Noralane Lindor, Verena Steinke-Lange, Elke Holinski-Feder, Jukka-Pekka Mecklin, Pål Møller

**Affiliations:** 1 Department of Tumor Biology, Institute of Cancer Research, Oslo University Hospital, Oslo, Norway; 2 Department of Gastrointestinal Surgery, Helsinki University Central Hospital, Helsinki, Finland; 3 Clinicum, University of Helsinki, Helsinki, Finland; 4 Division of Cancer and Genetics, Institute of Medical Genetics, Cardiff University School of Medicine, Cardiff, UK; 5 The Royal Melbourne Hospital, Melbourne, Australia; 6 University of Melbourne, Melbourne, Australia; 7 University of Manchester & Manchester University Hospitals Foundation Trust, Manchester, UK; 8 University of Newcastle and the Hunter Medical Research Institute, Callaghan, Australia; 9 University of Newcastle, Newcastle upon Tyne, UK; 10 University Witten-Herdecke, Wuppertal, Germany; 11 Department of Surgical Gastroenterology, Aalborg University Hospital, Aalborg, Denmark; 12 Central Finland Central Hospital, Education and Research, Jyväskylä, Finland; 13 Research Programs Unit, Genome-Scale Biology, University of Helsinki, Helsinki, Finland; 14 Karolinska Institutet, Stockholm, Sweden; 15 St Mark’s Hospital, Department of Surgery and Cancer, Imperial College London, London, UK; 16 University of Manchester and St Mary’s Hospital, Manchester, UK; 17 Hereditary Cancer Program, Catalan Institute of Oncology, Insititut d’Investigació Biomèdica de Bellvitge (IDIBELL), ONCOBELL Program, L’Hospitalet de Llobregat, Barcelona, Spain; 18 Centro de Investigación Biomédica en Red de Cáncer (CIBERONC), Madrid, Spain; 19 Department of Clinical Genetics, Vejle Hospital, Vejle, Denmark; 20 Department of Surgery, Regional Hospital West Jutland, Egtved, Denmark; 21 Department of Oncology, Aalborg University Hospital, Aalborg, Denmark; 22 Department of Pathology, Herlev Gentofte University Hospital, Herlev, Denmark; 23 High Risk and GI Cancer prevention Clinic, Gatro-Oncology Unit, The Department of Gastroenterology, Sheba Medical Center, Ramat Gan, Israel; 24 Leids Universitair Medisch Centrum, Leiden, Netherlands; 25 Center for Bioinformatics, Department of Informatics, University of Oslo, Oslo, Norway; 26 Department of Clinical Genetics, Aarhus University Hospital, Aarhus, Denmark; 27 Department of Applied Tumor Biology, Institute of Pathology, University Hospital Heidelberg, Heidelberg, Germany; 28 Cooperation Unit Applied Tumor Biology, German Cancer Research Center (DKFZ), Heidelberg, Germany; 29 Department of Health Sciences Research, Mayo Clinic, Scottsdale, AZ USA; 30 Medizinische Klinik und Poliklinik IV, Campus Innenstadt, Klinikum der Universität München, Munich, Germany; 31 MGZ- Medical Genetics Center, Munich, Germany; 32 Department of Surgery, Central Finland Central Hospital, Jyväskylä, Finland; 33 Faculty of Sport and Health Sciences, University of Jyväskylä, Jyväskylä, Finland

**Keywords:** Lynch syndrome, Survival, Colonoscopy, Surveillance, Cancer stage, Colon cancer

## Abstract

**Background:**

We previously reported that in pathogenic mismatch repair (*path_MMR*) variant carriers, the incidence of colorectal cancer (CRC) was not reduced when colonoscopy was undertaken more frequently than once every 3 years, and that CRC stage and interval since last colonoscopy were not correlated.

**Methods:**

The Prospective Lynch Syndrome Database (PLSD) that records outcomes of surveillance was examined to determine survival after colon cancer in relation to the time since previous colonoscopy and pathological stage. Only *path_MMR* variants scored by the InSiGHT variant database as class 4 or 5 (clinically actionable) were included in the analysis.

**Results:**

Ninety-nine *path_MMR* carriers had no cancer prior to or at first colonoscopy, but subsequently developed colon cancer. Among these, 96 were 65 years of age or younger at diagnosis, and included 77 *path_MLH1*, 17 *path_MSH2,* and 2 *path_MSH6* carriers. The number of cancers detected within < 1.5, 1.5–2.5, 2.5–3.5 and at > 3.5 years after previous colonoscopy were 9, 43, 31 and 13, respectively. Of these, 2, 8, 4 and 3 were stage III, respectively, and only one stage IV (interval 2.5–3.5 years) disease. Ten-year crude survival after colon cancer were 93, 94 and 82% for stage I, II and III disease, respectively (*p* < 0.001). Ten-year crude survival when the last colonoscopy had been < 1.5, 1.5–2.5, 2.5–3.5 or > 3.5 years before diagnosis, was 89, 90, 90 and 92%, respectively (*p* = 0.91).

**Conclusions:**

In *path_MLH1* and *path_MSH2* carriers, more advanced colon cancer stage was associated with poorer survival, whereas time since previous colonoscopy was not. Although the numbers are limited, together with our previously reported findings, these results may be in conflict with the view that follow-up of *path_MMR* variant carriers with colonoscopy intervals of less than 3 years provides significant benefit.

## Background

Initially, colonoscopy every third year was advocated to prevent death from colorectal cancer (CRC) in Lynch Syndrome (LS), whereas more recent clinical guidelines suggest colonoscopy at least once every 2 years, beginning between 20 and 25 years of age [1]. However, we have recently reported that for pathogenic mismatch repair (*path_MMR*) variant carriers, 1–2 yearly colonoscopy surveillance strategies do not result in a lower incidence of CRC, compared to a three-yearly strategy [2]. Recently, the Prospective Lynch Syndrome Database (PLSD) reported CRC stage data from 9 countries showing that 1–2 yearly colonoscopy was not associated with a diagnosis of CRC at an earlier stage than 3-yearly colonoscopy [2]. These findings were consistent with a report of no difference in CRC incidence and stage in LS patients between Germany (where colonoscopy surveillance is performed yearly), the Netherlands (1–2 yearly) or Finland (2–3 yearly) [3].

Geography and differences in follow-up practices might impact cancer risks, although no significant differences were identified in the most recently updated series of the PLSD study, which included 6350 *path_MMR* carriers who were prospectively observed for 51,646 follow-up years [4]. A recent single centre and retrospective French study suggested that an optimized colonoscopic surveillance program in LS patients might improve screening quality and possibly decrease CRC occurrence, but long-term prospective studies are needed to confirm these findings [5].

Because the current prospective evidence suggests that annual colonoscopies compared to three yearly colonoscopies do not benefit *path_MMR* carriers in terms of CRC incidence or earlier cancer stage at diagnosis, we aimed to determine if survival after colon cancer differs according to the intervals of colonoscopies undertaken prior to cancer diagnosis in *path_MMR* carriers. We also examined the relationship between stage at diagnosis and survival.

## Methods

### PLSD design

PLSD is an international, multi-centre database recording prospective observational data on *path_MMR* carriers under surveillance by colonoscopy [2, 6–10]. All collaborating centres undertook genetic testing with appropriate informed consents according to local and national requirements. No named data were exported to the PLSD.

### Inclusion criteria and statistical analysis

Inclusion criteria comprised only carriers with variants scored by the InSiGHT variant database (https://www.insight-group.org/variants/databases/) as class 4 and 5 (clinically actionable), and having a colon cancer detected during prospective follow-up. All cancers detected prior to, at or within 1 year after the age at the first planned and performed colonoscopy were scored as prior or prevalent cancers, and were excluded from the analysis when scoring prospectively observed cancers. Rectal cancer was not included because of known time-trends in outcome related to changes in management during the observation period and the low numbers of cases.

The following information was used in the statistical analyses: sex, *path_MMR* variant, age at inclusion, age at last update, age at death, age at colon cancer, type of cancer as indicated by the first three positions in the International Classification of Diseases version 9 (ICD-9) diagnostic system, the American Joint Committee on Cancer (AJCC) stage of colon cancer (I–IV) and the time since the last colonoscopy preceding the diagnosis of colon cancer. We considered AJCC stage III-IV as advanced [2].

The time elapsed since the colonoscopy before the one at which the cancer diagnosis was made was recorded and categorized, as previously reported [2]. Longer or shorter intervals between colonoscopies than planned may occur for several reasons in the clinical setting, and so we recorded actual time since last colonoscopy. We did not measure compliance with preset protocols, which would have been complex since protocols had changed during the observation period and different centres used different protocols at different times. We also compared time intervals and AJCC stage by MMR gene affected. Statistical testing was performed by SYSTAT13©.

### Survival analysis

Survival after colon cancer was estimated as previously described [6, 7, 9]. Briefly, the Kaplan-Meier survival function from first prospectively detected colon cancer diagnosis to death was calculated. To minimize probabilities of death from other causes, survival calculations were restricted to cases diagnosed with colon cancer at 65 years of age or younger.

## Results

Ten centres in nine different countries (Finland, Sweden, Norway, Denmark, The Netherlands, UK, Spain, Israel and Australia) contributed to this study. Together, they reported 196 *path_MMR* carriers without prior or prevalent colon cancer at inclusion having had prospectively detected colon cancer. Ten of these had had two prospectively detected colon cancers and were excluded, leaving 186 cases with one prospectively detected colon cancer for survival analysis. Among these, 96 *path_MMR* carriers having had no prior or prevalent cancer in any organ were later prospectively diagnosed with colon cancer at age 65 years or younger. These 96 *path_MMR* carriers were selected for survival analysis and included 77 *path_MLH1*, 17 *path_MSH2* and 2 *path_MSH6* carriers.

The numbers of colon cancers detected within < 1.5, 1.5 to 2.5, 2.5 to 3.5 and > 3.5 years since previous colonoscopy were 9, 43, 31 and 13, respectively. The median time since previous colonoscopy to colon cancer was 28 months (2.3 years). Only one patient was diagnosed with a stage IV disease (*path_MLH1* carrier with a 2.5 to 3.5 years interval, who died within 5 years of diagnosis). Ten *path_MLH1* carriers, 7 *path_MSH2* carriers, and none of the *path_MSH6* carriers were diagnosed with a stage III colon cancer (Table 1). Data on gender and colonoscopy interval broken down by gene are presented in Table 2.

**Fig. 1:**
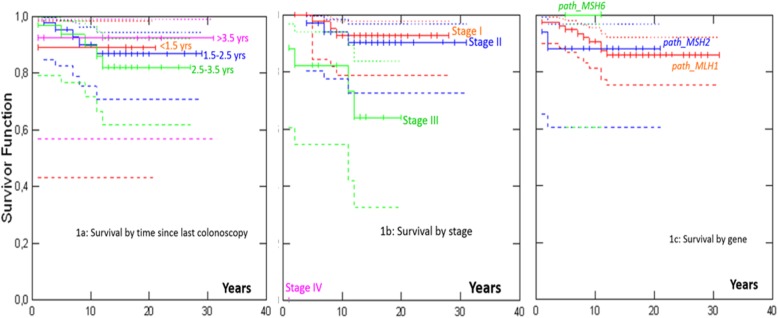
Survival after colon cancer in 96 *path_MMR* carriers under 65 years of age without prior or prevalent cancer in any other organ

Five- and 10-year crude survival after colon cancers that were diagnosed before 65 years of age in *path_MLH1*, *path_MSH2* and *path_MSH6* carriers are presented in Table 3 and Fig. 1. Median follow-up time after prospectively detected colon cancer was 14 years. Ten-year survival when the last colonoscopy had been undertaken < 1.5, 1.5–2.5, 2.5–3.5 or > 3.5 years before diagnosis, was 89, 90, 90 and 92%, respectively (*p* = 0.91). Ten year crude survival was 93% for stage I, 94% for stage II and 82% for stage III (*p* < 0.001). There were no significant differences in survival according to which MMR gene was involved (*p* = 0.90).

**Table 1:** Number of cases by gene and stage

CRC Stage	*path_MLH1*	*path_MSH2*	*path_MSH6*
Stage I	37	7	1
Stage II	29	3	1
Stage III	10	7	0
Stage IV	1	0	0

**Table 2:** Gender and colonoscopy interval distribution by *path_MMR* gene of the 96 *path_MMR* carriers included in the study

Parameter	*path_MLH1*	*path_MSH2*	*path_MSH6*
Gender			
Female	41	9	2
Male	36	8	0
Colonoscopy interval			
Less than 1.5 years	7	2	0
1.5 to 2.5 years	32	10	1
2.5 to 3.5 years	29	1	1
Over 3.5 years	9	4	0

**Table 3:** Crude survival (%) after selected colon cancer diagnosed after initiation of colonoscopy surveillance and before age of 65 years for *path_MLH1*, *path_MSH2* and *path_MSH6* carriers with no cancer in any organ prior to, at, or within 1 year of their first colonoscopy

Parameter	5-year survival (%)	95% CI (%)	10-year survival (%)	95% CI (%)
*Interval*				
Less than 1.5 years	89	[43–98]	89	[43–98]
1.5 to 2.5 years	95	[83–99]	90	[75–96]
2.5 to 3.5 years	94	[77–98]	90	[72–97]
Over 3.5 years	92	[57–99]	92	[57–99]
*Stage* ^*a*^				
I	98	[84–99.7]	93	[79–98]
II	97	[80–99.6]	94	[78–98]
III	82	[55–94]	82	[55–94]

^*a*^IV: One *path_MLH1* carrier who died before 5 years

**Fig. 2:**
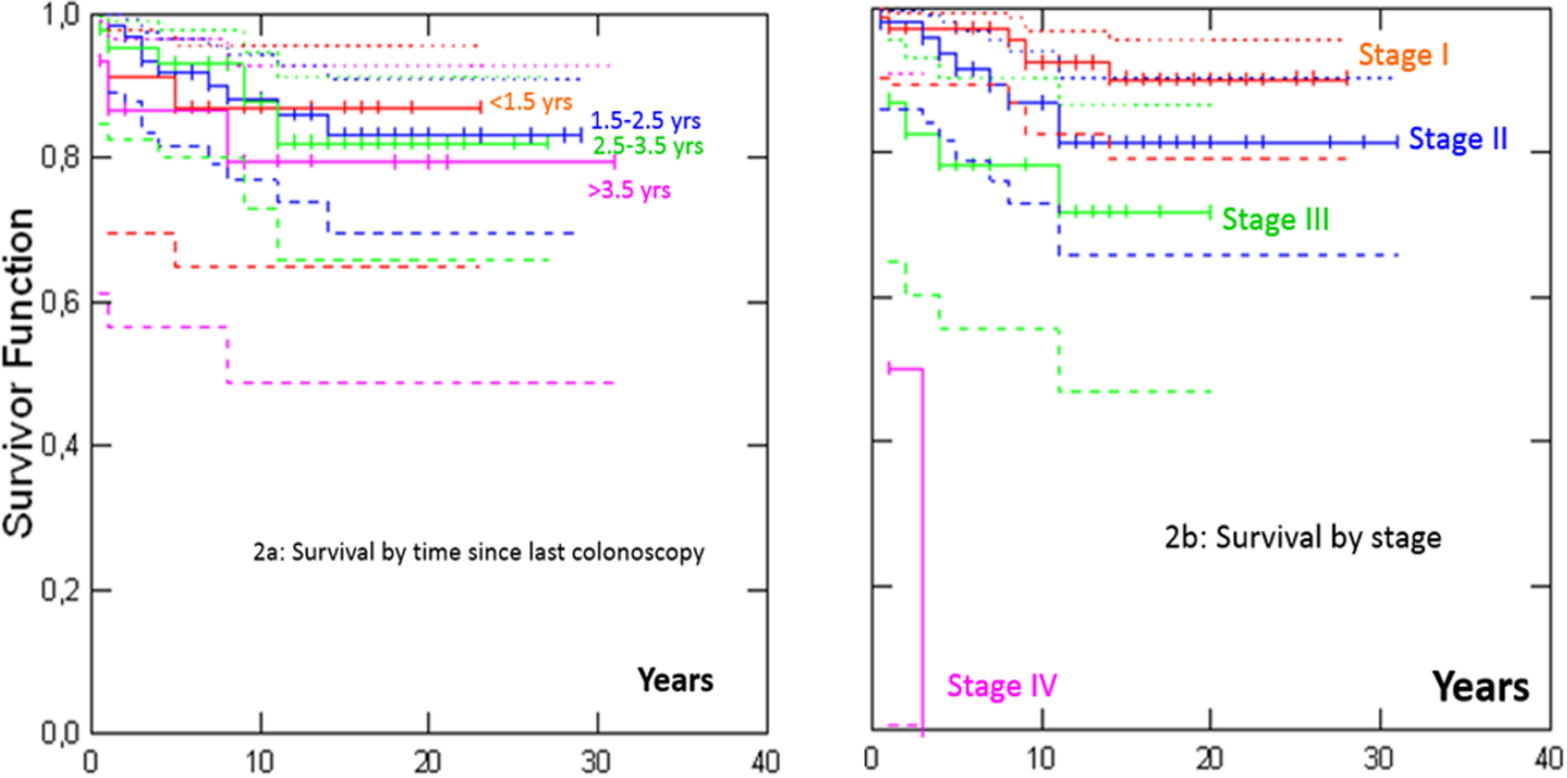
Survival after colon cancer in 143 *path_MMR* carriers under 65 years of age with or without prior or prevalent cancer in any other organ

We also performed a sensitivity analysis determining survival according to the time since last colonoscopy by including *path_MMR* carriers with all prospectively detected colon cancers (i.e. also including those patients with prior or prevalent cancer in other organs, of whom 143 had colon cancer diagnosed at 65 years of age or younger), and arrived at similar results to those for the 96 *path_MMR* carriers without any prior or prevalent cancer (Fig. 2) i.e. no significant association with time since last colonoscopy (*p* = 0.93); and significant association with stage (*p* < 0.001).

## Discussion

In the current study, we found no demonstrable difference in crude survival after colon cancer in *path_MMR* carriers who had had their last colonoscopy 3 years before the diagnosis of colon cancer, compared to those with shorter times between last colonoscopy and diagnosed colon cancer. Considered together with our previous reports, in which we found no decrease in the incidence of CRC with advanced stages of CRCs where the interval since last colonoscopy was less than 3 years before cancer diagnosis, our findings may be considered to be in conflict with the view underlying the current recommendations on the follow up of *path_MMR* carriers with colonoscopy more often than every 3 years.

In a previous report, we discussed the possibility that not only microsatellite unstable non-invasive lesions, but also infiltrating cancers in LS, may be removed by the host immune system [2]. Aysel Ahadova et al. suggested that although some CRCs in LS may develop from MMR-proficient adenomas after secondary inactivation of the MMR system, a larger portion of LS CRCs appear to develop from MMR deficient crypt foci, either through an adenomatous phase or as non-polyp lesions with immediate invasive growth that may not have colonoscopically visible precursor lesions [11, 12]. Our current report does not aim to provide a full discussion of the biological models that may explain our results: the few we mention are provided to illustrate the wide range of possible explanations that require further investigation.

Our study included only *path_MLH1* and *path_MSH2* carriers, except for two *path_MSH6* carriers, and any conclusions based on this report should be applied to *path_MLH1* and *path_MSH2* carriers only. Besides, it has already been described that *path_PMS2* carriers have almost no incident cancer risk under surveillance.

Clinical guidelines should be based on observed outcomes of interventions. Whatever the reasons for the results presented herein, we report our observed prospective empirical outcomes of clinical guidelines that aim to prevent colon cancer in *path_MMR* carriers. Our data may therefore be useful in formulating future revisions to clinical guidelines for LS.

In our study, the survival analyses included only colon and not rectal cancers. There were several reasons for this, including differences in the treatment, classification and time from diagnosis to surgical resection of rectal cancers (e.g. this can vary from immediate to delays of several months due to neoadjuvant therapy). In addition, wide variation in the treatment choices across different regions/countries have been described [13]. Since these differences may be confounders to our chosen endpoint, survival by stage, we decided to perform survival analysis only in colon cancers. It is, however, of utmost importance to also report these measures for rectal cancer separately. To achieve this, we will need to collate data for more rectal cancer cases than are currently reported to the PLSD.

There are several limitations to the current study. Numbers included are limited. All centres previously having contributed to the PLSD reports were invited to participate, but not all provided data to this report. We present no information on survival after a second colon cancer subsequent to a first colon cancer. It is possible that some existing lesions were missed during earlier colonoscopies that preceded the diagnosis of colon cancers. We have not excluded that colonoscopy with new techniques and better knowledge on what to look for may prevent cancer and improve survival, but even if so it still remains to be demonstrated that such is possible to implement in a broader health care setting: what we report is the observed outcome of health care so far, not what putatively might have been obtained otherwise. However, if colonoscopy quality did play a significant role related to survival, the impact might be expected to be greatest for the lesions missed with a three-yearly strategy, and survival would be expected to be worse in that group. Because we have recently shown that there is no significant difference between AJCC stages of cancers diagnosed when colonoscopies are done yearly or three-yearly, and as annual colonoscopy has been implemented more recently, there is no reason to assume that colonoscopies with short intervals were of lower quality. We restricted our analysis to the core subset of 96 carriers described to provide a robust crude survival analysis for colon cancer in *path_MMR* carriers with as few confounders as possible, and we found the same results when also including cases with prior or prevalent cancers in other organs.

While we report the largest prospective analysis undertaken so far of colon cancer survival in LS patients according to time since last colonoscopy, we do not, based on the current and our previous reports, advocate any revision of current clinical guidelines, and the PLSD will ask more contributors to provide data to repeat this study in an independent data set. More detailed studies are needed to understand why there are still some carriers dying from colon cancer, and how this may be prevented. Health economic studies are indicated to establish whether colonoscopy more frequently than every 3 years is justified from a resource perspective. These studies require data on the costs from all aspects, including unwanted (side-) effects, borne by health care systems and patients. The PLSD is presenting some of the many pieces of evidence that need consideration in discussions to revise current clinical guidelines for LS surveillance.

## Data Availability

The datasets used and/or analysed during the current study are available from the corresponding author on reasonable request. We have published a website www.lscarisk.org on which cancer risks for all published data can be reviewed and calculated in graphic form.

## Abbreviations

AJCC: American Joint Committee on Cancer
CRC: colorectal cancer
LS: Lynch syndrome
*path_MLH1*: Pathogenic (disease-causing) variant of the MLH1 gene
*path_MMR*: Pathogenic (disease-causing) variant of any mismatch-repair gene
*path_MSH2*: Pathogenic (disease-causing) variant of the MSH2 gene
*path_MSH6*: Pathogenic (disease-causing) variant of the MSH6 gene
*path_PMS2*: Pathogenic (disease-causing) variant of the PMS2 gene
